# Will They Stay or Will They Go? International Graduate Students and Their Decisions to Stay or Leave the U.S. upon Graduation

**DOI:** 10.1371/journal.pone.0118183

**Published:** 2015-03-11

**Authors:** Xueying Han, Galen Stocking, Matthew A. Gebbie, Richard P. Appelbaum

**Affiliations:** 1 Center for Nanotechnology in Society, University of California Santa Barbara, Santa Barbara, CA, United States of America; 2 Department of Political Science, University of California Santa Barbara, Santa Barbara, CA, United States of America; 3 Materials Department, University of California Santa Barbara, Santa Barbara, CA, United States of America; 4 Global & International Studies, University of California Santa Barbara, Santa Barbara, CA, United States of America; Universidad Veracruzana, MEXICO

## Abstract

The U.S. currently enjoys a position among the world’s foremost innovative and scientifically advanced economies but the emergence of new economic powerhouses like China and India threatens to disrupt the global distribution of innovation and economic competitiveness. Among U.S. policy makers, the promotion of advanced education, particularly in the STEM (Science, Technology, Engineering and Mathematics) fields, has become a key strategy for ensuring the U.S.’s position as an innovative economic leader. Since approximately one third of science and engineering post-graduate students in the U.S. are foreign born, the future of the U.S. STEM educational system is intimately tied to issues of global competitiveness and American immigration policy. This study utilizes a combination of national education data, a survey of foreign-born STEM graduate students, and in-depth interviews of a sub-set of those students to explain how a combination of scientists’ and engineers’ educational decisions, as well as their experience in school, can predict a students’ career path and geographical location, which can affect the long-term innovation environment in their home and destination country. This study highlights the fact that the increasing global competitiveness in STEM education and the complex, restrictive nature of U.S. immigration policies are contributing to an environment where the American STEM system may no longer be able to comfortably remain the premier destination for the world’s top international students.

## Introduction

In his 2012 State of the Union address, U.S. President Barack Obama decried the fact that foreign-born students are at risk of forced deportation, potentially robbing the American economy of its innovation and expertise:

Let’s also remember that hundreds of thousands of talented, hardworking students in this country face another challenge: the fact that they aren’t yet American citizens. Many were brought here as small children, are American through and through, yet they live every day with the threat of deportation. Others came more recently, to study business and science and engineering, but as soon as they get their degree, we send them home to invent new products and create new jobs somewhere else….That doesn’t make sense. …. let’s at least agree to stop expelling responsible young people who want to staff our labs, start new businesses, defend this country. Send me a law that gives them the chance to earn their citizenship. I will sign it right away [1].

Yet this concern obscures many of the global educational and regulatory trends that are re-shaping innovation in high-tech industries like nanotechnology. Approximately one third of science and engineering post-graduate students in the United States are foreign-born, with particular concentrations in computer science and physics [2]. These numbers dropped precipitously after 9/11, as American immigration policy shifted to be more restrictive and other countries improved their university systems [3], although this trend seems to have leveled off since 2010 [2]. Moreover, upon graduation, many students are drawn back to their birth countries, which seek to offset ‘brain drain’ problems through encouraging expats—particularly those in STEM (Science, Technology, Engineering, and Mathematics) fields—to return. A decline in the number of foreign-born students, scientists and engineers can hamper the United States’ innovation capacity [4, 5].

This study investigates how these new opportunities have shaped students’ choices as they select graduate programs and plot their post-graduate career. Our inquiry also examines the influence of the graduate student’s education in the U.S. in shaping his or her career path. Through the combination of national data, an on-line survey, and in-depth interviews of foreign-born students in science and engineering fields at a highly ranked research university in California, we explain how an integration of scientists’ and engineers’ educational decisions as well as their experience in school can predict a student’s career path and location. This can have considerable policy implications and affect the international innovation balance.

## Conceptual Framework

Scholars and policymakers have studied the ‘brain drain,’ or the migration of skilled workers from developing countries to developed countries, since at least the 1960s [6, 7]. Brain drain literature is concerned with the economic and development impact caused by this migration, which early models showed to be a net negative for the sending country and a net positive for the receiving country [8]. Yet these models did not account for the potential of remittances [9], intergenerational transfer of skills [3, 10, 11], and skill spillover to other workers [12, 13], as well as the diffusion of innovative technologies from developed states to offset this negative [4, 14, 15]. In particular, advancements in information technology and the increasing interconnectedness of a globalized economy have created new opportunities for migrants to quickly push innovations back to their home countries [6, 7, 16]. These returning students are often thought to be more entrepreneurial than those who stayed [8, 17, 18]. The success of these migrants in creating new industries in their home countries have prompted many states with high skilled migrant outflows to create new opportunities for these migrants to participate in the local economy [9, 19, 20], and, in some cases, return home [13].

In the American context, many of the most innovative scientists and engineers migrated to the United States to pursue an education [3, 11]. In fact, approximately 40% of science and engineering post-graduate students in the United States are foreign-born, yet this rate is dropping as the combination of stricter immigration laws and the maturation of university systems abroad have lured students elsewhere [3, 21]. The largest source country is China, while the United States is the most popular destination country [11].

Several scholars have developed models to explain why students migrate. One of the most widely utilized of these models explains this decision making process as a series of ‘push-pull’ factors that influence students’ decisions. Push factors are characteristics of the home country that compel a student to study abroad, particularly economic or social factors that limit educational opportunity. Pull factors refer to perceived benefits of the destination country or institution and include personal recommendations, cost, the overall environment, geographic proximity, and social links [22]. Additionally, reverse pull factors, such as family links and the improvement of domestic institutions can limit the effectiveness of these push-pull factors. Such models have also been extended to incorporate the socioeconomic and personal characteristics of the student him/herself, including personal aspirations and academic ability [23]. On the other hand, some studies avoid using the push-pull framework precisely because it has little to say about influences at the individual level [24]. Instead, such studies emphasize the importance of networks to explain the ins and outs of studying abroad or information from peers or influencers about the experience [25].

As described above, once students attend school abroad, they tend to transfer some of their skills back to their home country, whether in the form of actual skill transfer [10, 12] or through entrepreneurial activity [17–18]. A number of factors could drive a student’s decision to return to his or her home country or to stay in the country in which he or she studied. Bratsberg proposed a simple economic model, in which an individual will choose to live wherever his or her skills will be most valued, although political conditions in the home country will limit that consideration substantially [26]. Others have suggested that networks play a key role: successful returnees reach out to their peers and friends who have not returned, informing them of opportunities and creating a social network. In Taiwan, for instance, this led to several distinct behaviors: initial returnees, who fostered an innovative environment; individuals in their networks who joined them and also recruited from their networks; and temporary returnees, who work in both Taiwan and the destination country and reinforce the links between them [27].

Others have suggested that lifestyle is more important in this decision. Migrants who still have strong ties to their families in their home country are more likely to return home [28]. Students may also be drawn home for social and cultural factors, such as difficulty integrating into the destination’s social or academic culture. In some cases, this can be the result of discrimination suffered as a consequence of their nationality [29].

Given the real (and perceived) economic benefits of those with higher education, particularly in STEM fields, source countries therefore have an incentive to retain the brightest students as well as convince those who have gone abroad to return home. Some of these latter programs, like the “Young Talent Program” in Brazil [30], fund students to study abroad with the requirement that they return after completing their studies. On the other hand, many countries already have large populations studying abroad, so they offer tax breaks, grants, and other incentives to convince ex-patriots to return. A summary of relevant programs can be found in Table 1.

**Table 1:** List of programs, by country, that promotes the return of Science, Technology, Engineering, and Mathematics (STEM) talent back to their home country.

Country	Program Name	Website	Program Description
Argentina	R@ICES	www.raices.mincyt.gov.ar	A program under the Ministry of Science, Technology and Productive Innovation of Argentina. The goals of the program are to strengthen the link between Argentine researchers in the country and abroad, bring Argentines abroad back to Argentina to develop research, and implement retention policies that promote the return of Argentines.
Bavaria	Return to Bavaria	www.returntobavaria.com	Sponsored by the Bavarian Ministry of Economic Affairs and Media, Energy and Technology, the program was initiated in 2012 to motivate Bavarian and German professions to return home.
Brazil	Science Without Borders “Young Talent Program” (i.e., Jovens Talentos)	www.cienciasemfronteiras.gov.br	A joint effort from Brazil’s Ministry of Education and the Ministry of Science and Technology, the program aims to (1) place 100,000 Brazilian students and researchers in top universities worldwide by 2014 and (2) to attract talented young researchers from outside the country, especially Brazilians, to Brazil.
Chile	Start-up Chile	startupchile.org	Program started by the Chilean government in 2010 to attract early stage entrepreneurs to build their startup companies in Chile.
China	1000 Talents Program	www.1000plan.org	Launched by the Central Organization Department of the Chinese Communist Party in 2008, the program aims to recruit 1000 outside Chinese talents to return to China.
Europe	Horizon 2020	ec.europa.eu	Commencing in 2014, Horizon 2020 is an initiative aimed at securing Europe’s global competitiveness. There are many different programs (e.g., European Research Council Starting Grants, European Research Council Advanced Grants, Marie Sklodowska-Curie Actions Program, etc.) that facilitate the return of young European scientists back to Europe.
Germany	German Academic International Network (GAIN)	www.gain-network.org	Created by the Deutscher Akademischer Austausch Dienst (i.e., German Academic Exchange Service) in cooperation with the German Research Foundation and the Alexander von Humboldt Foundation, the program provides support, networking opportunities, workshops, and job postings for German scholars and scientists working in North America. GAIN promotes the dissemination of information across the Atlantic and prepares German scientists to return to Germany.
Israel	Gvahim	gvahim.org.il	Initiated in 2006, this non-governmental organization promotes Israel’s “Brain Bain” efforts by offering highly-skilled Olim with opportunities and networking in Israel.
Italy	Dulbecco Telethon Institute	dti.telethon.it	Founded in 1999, the institute provides funding to early stage researchers who work on human genetic diseases.
Moldova	Gsorm Gala Studenilor	galastudentilor.md	Moldovan students abroad competed in the competition “Academic Excellence Moldova”. The program encourages Moldovan students abroad to return to Moldova.
Portugal	Cienca 2007	www.fct.pt	An international call for 1000 post-doctoral research positions, both Portuguese and foreign nationals, at Portuguese scientific institutions. The program was launched and closed in 2007.
Russia	Mega Grant (i.e., Resolution No. 220)	www.p220.ru	Launched in 2010 by the Government of the Russian Federation, the program provides grants of up to $5 million USD to conduct research in Russia. The program hopes to bring Russian scientists residing abroad as well as foreign scientists to Russian institutions.
South Korea	Brain Return 500*	www.ibs.re.kr/en/careers/brainReturn.jsp	Established by the Institute for Basic Science, the goal of the program is to attract 500 talented young scholars and scientists back to South Korea by 2017.
Spain	Spanish Ramón y Cajal Program	www.mineco.gob.es	Funded by the Spanish Ministry of Economy and Competitiveness, the program provides financial support to PhD researchers for a period of five years.
Sub-Saharan Africa	Homecoming Revolution	homecomingrevolution.com	Started in 2003, the goal of Homecoming Revolution is to bring highly skilled Africans back to their homelands.
Sweden	Study in Sweden Swedish Institute	www.studyinsweden.se	The institute is a public agency that provides grants to researchers around the world in order to establish cooperating and lasting relations with other countries. A variety of programs and grants are available depending on the applicant’s nationality.
Thailand	Reverse Brain Drain (RBD)	www.nstda.or.th	The RBD initiative by Thailand’s National Science and Technology Development Agency began in 1990. Initially, the primary goal of the initiative was to promote the permanent return of overseas Thai professionals. In 1997, the RBD’s main objective shifted to the promotion of temporary returns of science and technology professionals. As of 2007, RBD promotes the brain circulation of Thai professionals overseas.
Turkey	2232 Repatriation Research Scholarship Program	www.tubitak.gov.tr	Enacted by the Scientific and Technological Research Council of Turkey, the program encourages the return of successful Turkish researchers from abroad to continue their work in their home country.

Since the brightest students therefore have numerous opportunities, how do they decide which ones to pursue? We hypothesize that their motivation for the initial decision to either remain in their home country or study abroad will be a result of an analysis of the quality of education they can receive in their home country compared to their perception of the quality of education in a destination country. This will be mediated by personal factors, including socioeconomic factors, personal ambition, and connection to family. We further hypothesize that those who study abroad are imbued with personal ambition, and, upon graduation, will seek to go where their skills will be most valued, although this ambition will be internally moderated by a desire to return to their families. For those who are not inclined to return home, their decisions will be limited by the frictions of career choice, immigration restrictions, and personal interactions.

A parallel question regards the initial career choices of graduating doctoral and masters students: while many may have entered graduate school with academic aspirations, there are also attractive options for a PhD in STEM fields in industry that may prove decisive upon graduation. Additionally, there are geographic and other restrictions on a career in the academy that are not as prevalent in the business community.

Unlike other studies, which either analyze each factor discretely or group them into personal, social, and professional factors, we hypothesize that the greatest insight can be afforded by observing the interactions of these factors. Accordingly, a person may be interested in going into academia after he or she graduates, but the impact of a factor, such as the strength of one’s network, is not independent of other factors, like one’s adjustment to life in the U.S. or treatment in graduate school. Consequently, we propose an interactive model, in which we identify the interdependencies between each factor. This allows us to build a decision tree that identifies the most important factors as well as how they interact with each other. How those factors influence each other is further dependent upon the individual’s experience.

Through a survey and follow up interviews, we examine three decisions through this interdependency model. For the first decision—whether to pursue higher education on one’s home country to come to the United States to study—we draw on previous research, identifying the following factors as potentially important: quality of education, career opportunities, experience living abroad, opportunity to work with specific faculty, a desire to live in the United States, and proximity to friends or family. For the second and third decisions—to remain in the United States upon graduation or return home, and to pursue a career in academia or industry—we hypothesize that these decisions are influenced both by professional factors (career plans, treatment in school, job opportunities, quality of advising, professional networks), personal factors (parents’ education, socioeconomic status), and social factors (desire to live in the United States, adjustment to American society, be close to friends and family).

## Methods

We combine the results of three separate measures to reach our conclusions. For national and historical data, we used data from the Institute of International Education’s national surveys [31]. We supplemented this national data with survey data at the University of California—Santa Barbara, a Research 1 Institution with highly-ranked programs in STEM fields. To provide greater clarity, we conducted follow up interviews with volunteer respondents to this survey. This hybrid approach afforded us the broad scope of national data while leading us to some of the in-depth findings of a more limited survey.

### International students in the U.S. over time

The Institute of International Education has been tracking the number of international students studying at higher education institutions in the U.S. since 1949 and the data have been published annually in its *Open Doors Reports* (www.iie.org). Data are collected annually through surveys sent to approximately 3,000 accredited U.S. higher education institutions. These institutions provide voluntary information regarding the international students enrolled at their respective campuses. The reports provide comprehensive data on international students’ places of origin, sources of financial support, fields of study, host institutions, academic level, rate of growth of international students in the U.S., and the economic impact of international students to the U.S. economy.

### Survey design and implementation

The primary goals of our study were threefold: to determine which factors influenced foreign STEM students to pursue their education in the United States; their plans to remain or return home after graduation; and their decision to pursue academic or business-oriented careers. To accomplish this, we emailed international graduate students in STEM fields at the University of California, Santa Barbara (UCSB) with an introduction to the study and a survey link, assuring anonymity for their responses. UCSB is a public research university with a total enrollment of approximately 22,000 students, of which about 3,000 students are those at the graduate level. Human Subjects approval for the survey and follow-up interviews was granted by the University of California, Human Subjects Committee, Office of Research, Santa Barbara. Participants provided consent to participate in the survey via an online agreement. Individuals who participated in follow-up interviews also provided written consents. Responses were confidential and were not associated in any way with a participant's identity. Data presented in this manuscript are aggregated such that responses are anonymized. The survey consisted of four categories of questions: (1) basic background information (*e.g*., age, gender, major, year of study); (2) reasons for studying in the U.S.; (3) perceptions of their graduate education in the U.S.; and (4) plans after graduation. The online survey was active from May 2–24, 2013, and resulted in a 42% completion rate (the full survey can be found as S1 Survey). Students were given an option at the end of the survey to specify their interest in conducting any follow-up interviews. A total of 12 follow-up interviews were conducted between January 27 and February 21, 2014. See http://dx.doi.org/10.6084/m9.figshare.1121633 for raw survey data.

### Data processing and statistical analysis

A total of 166 international graduate students, representing 32 different countries, responded. We used a classification tree to determine how professional, social, and personal factors interact to influence students’ decisions on choosing to stay or depart from the U.S. upon graduation. Classification tree models provide a robust method to deal with nonlinear relationships, high-order interactions, and missing values (for reviews, see [32–34]). A classification tree is a nonparametric regression approach in which models are obtained through recursive partitioning of the data space. The data space in this study is the space spanned by all predictor variables that may influence whether an individual will stay or depart the U.S. upon graduation. Observations with similar response values are grouped within a partition. Once a classification tree is fitted under the full model, the tree is ‘pruned’ to avoid overfitting of the model. Pruning occurs by eliminating branches that do not add to the prediction accuracy in cross validation. The classification tree in this study was fitted using the ‘rpart’ package in R [35] and pruned to a seven-leaf tree using the cross-validation method with the 1-SE rule. We downloaded all survey responses and used R [36] for all data cleaning, standard statistical summarizations, and statistical analyses.

### Foreign programs to attract expatriates

To determine the extent to which brain drain is viewed as a problem that countries are seeking to address, we identified programs around the globe that are aimed at attracting expatriates back to their home countries. We used a web-crawling approach using search terms “reverse brain drain + [country name]” and “bring researchers back to [country name]” to find these programs.

## Results

### International students in the U.S. over time

**Fig 1:**
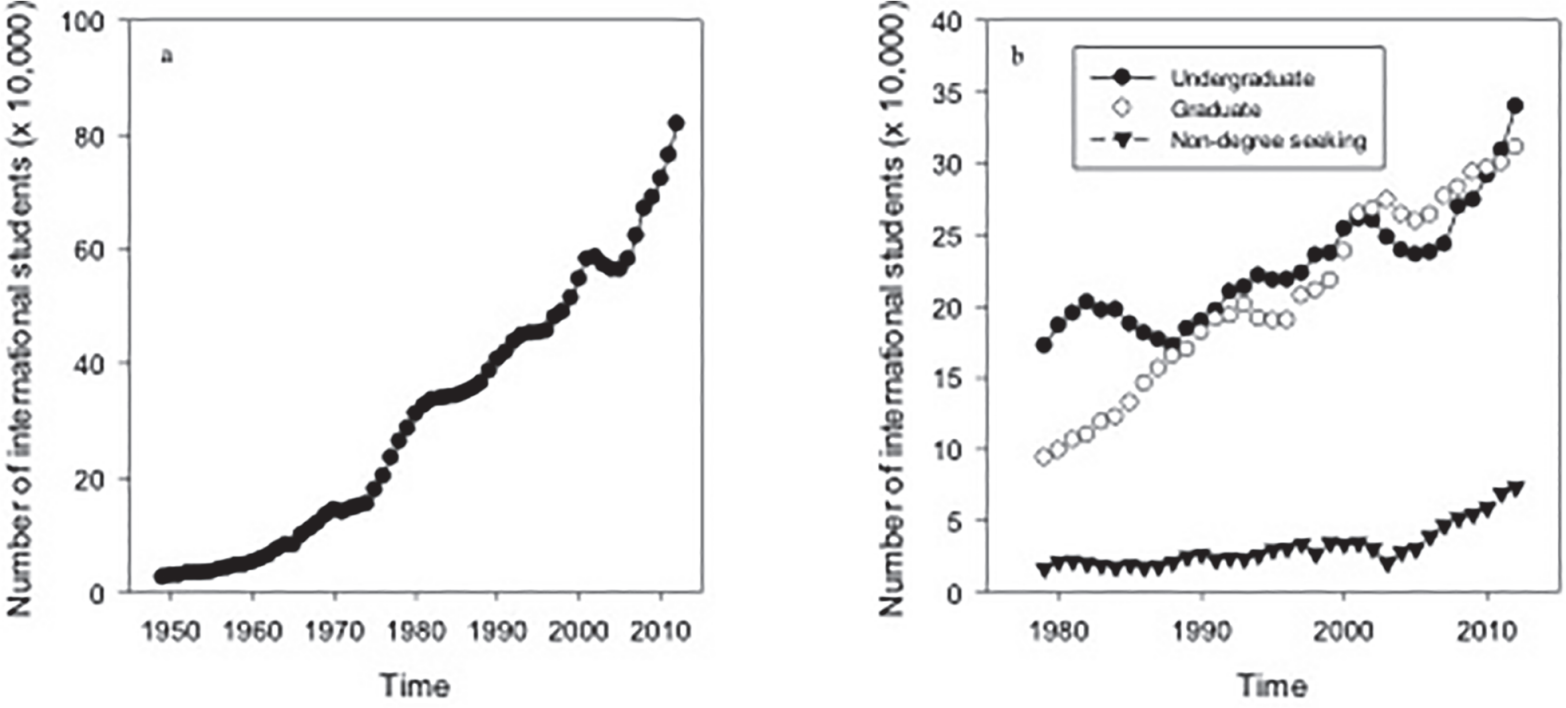
Temporal trends of international students studying in the U.S. (a) Total number of international graduate students studying in the U.S. from 1949/50–2012/13. (b) Breakdown of international students studying in the U.S. by academic level from 1979/80–2012/13. Source: Institute of International Education, Open Doors Reports.

The total number of international students studying in the U.S. has steadily increased since 1949/50 (Fig. 1A). In 2011/12, Asia was the leading place of origin and accounted for 64% of all international students studying in the U.S. followed by Europe (11%), Latin America (8.4%), the Middle East (7.5%), Africa (4.6%), North America (3.6%), and Oceania (0.75%). China was the leading country of origin, accounting for 28.7% of all international students in 2012/13, followed by India (11.8%), South Korea (8.6%), Saudi Arabia (5.4%), and Canada (3.3%). The number of international undergraduate students studying in the U.S. has experienced an average (± 1 SE) of 7.0 (± 1.6%) increase in the past five years while international graduate students have only increased by 2.5% (± 0.57%) over the same time period. The number of international undergraduate students has been consistently higher than the number of international graduate students studying in the U.S. with the exception of 2001/02–2010/11 (Fig. 1B). Graduate students accounted for 46% of the total number of international students in 2002/03 but only 38% of the total international student population as of 2012/13. During the same time period, undergraduate students accounted for 44% in 2002/03 and 42% in 2012/13. During the same period the number of non-degree seeking international students has more than doubled from ∼20,000 students in 2002/03 to ∼74,000 in 2012/13, to account for 5.2% of all international students in 2002/03, increasing to 8.9% ten years later. The percentage of international students studying in a STEM field (36.7%) was more than double that of those studying in a social science or humanity discipline (16.6%) in 2012/13.

### Survey

Our study received 166 completed survey questionnaires from students representing 32 different countries (S1 Summary). Despite having a small sample size, the demographics of our survey respondents matched closely to that of the national distribution of international students studying in the U.S. Asia was the leading place of origin among survey respondents and accounted for 69.2% (64%) of the survey (national) population of international students, followed by Europe at 12.4% (11%), the Middle East at 9.5% (7.5%), Latin America at 4.7% (8.4%), North America at 2.4% (3.6%), Africa at 1.2% (4.6%), and Oceania at 0.6% (0.75%). The largest sending country was China, accounting for 28% of all respondents, followed by India (24%), Taiwan (7%), South Korea (5%), Iran and Turkey (4% each). Of the total number of respondents, a quarter were female and three-quarters male. Master’s level and PhD level students accounted for 21% and 79%, respectively, of our survey respondents. Engineering students accounted for 73% of our respondents and life and physical sciences accounted for the remaining 27%.

### Reasons for coming to the U.S.

**Fig 2:**
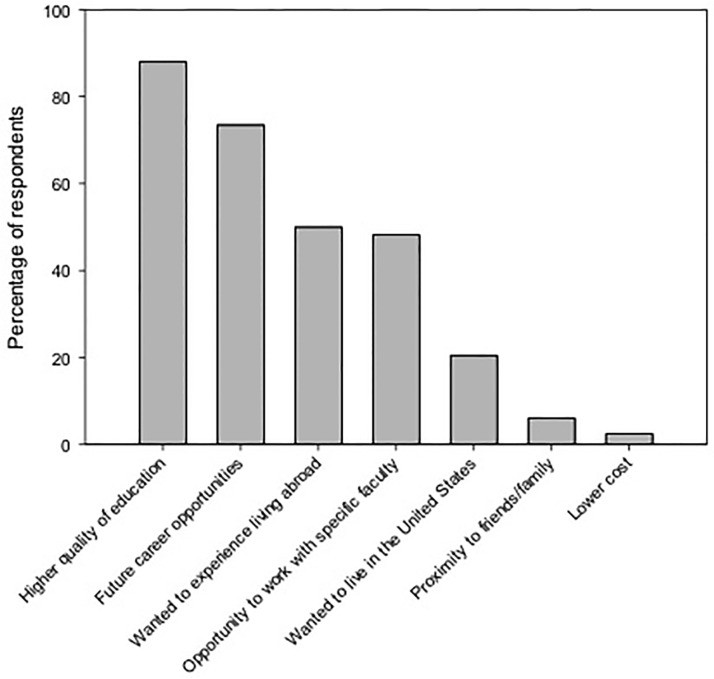
Reasons for studying in the United States. Percentage of respondents that selected each factor as an influential reason behind their decision to conduct their graduate studies in the United States. Respondents were allowed to select multiple factors.

Students indicated that professional factors were more important than social and personal reasons in their decisions to conduct graduate studies in the U.S. (Fig. 2). Higher quality education and future career opportunities were the top two factors that influenced students’ decisions to study in the U.S. (88% and 74%, respectively; Fig. 2). On a scale from 1 (low) to 5 (high), students strongly believed that their U.S. education will provide them with a strong advantage in their careers (mean = 4.36, SE = 0.07). Large percentages of respondents believed that in comparison with their home country, a U.S. education provides better education/knowledge of their chosen field (83%), better professional network (73%), better advisors/mentorship (70%), and better job opportunities (69%).

Students on average, on a scale of 1 (have not adjusted well to American educational culture) to 5 (have adjusted well to American educational culture), felt that they have adjusted well to the American educational culture (mean = 3.95, SE = 0.06). During their adjustment period, students indicated that they encountered a variety of challenges (cultural: 62%, social: 51%, financial: 40%, academic: 35%, and racial: 10%).

### Factors for staying/departing the U.S. upon graduation and career plans

**Fig 3:**
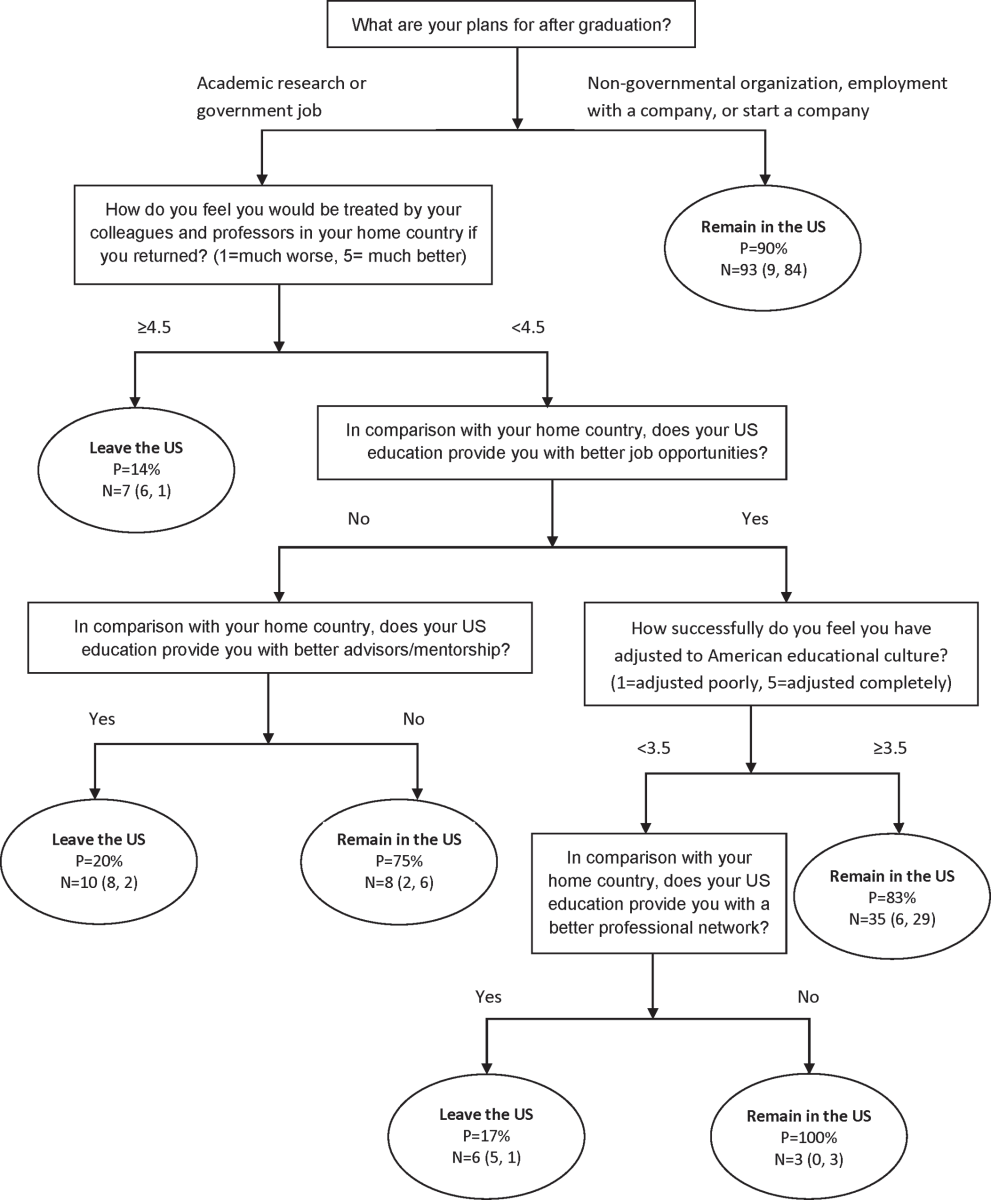
Decision Tree—Reasons for remaining in the U.S. or leaving upon graduation. Final classification tree of factors influencing whether international graduate students will remain in the US upon graduation after pruning using the cross-validation with one standard error rule. ‘P’ is the fitted probability of students staying in the US after graduation. ‘N’ provides the number of students for that terminal leaf of the tree. The numbers in parentheses following the sample size are the number of students who chose to leave the US and the number of students who would like to remain in the US upon graduation, respectively, for each terminal leaf of the tree.

**Fig 4:**
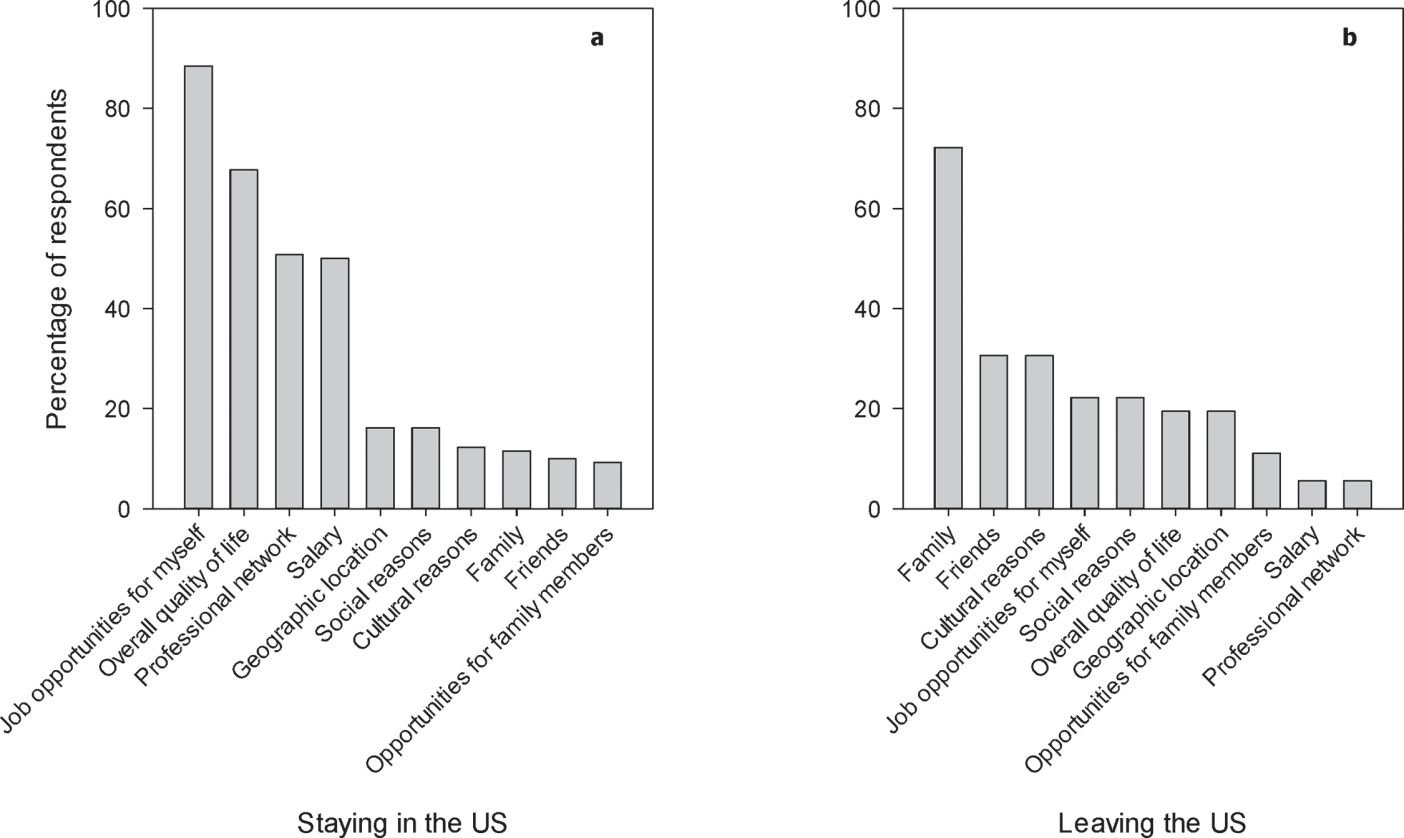
Reasons given for remaining in or leaving the United States. Percentage of respondents that indicated each factor as an influential reason behind their decision to (a) remain in the US among students who wish to remain in the US upon graduation (n = 130); (b) to leave the US among students who wished to leave the US upon graduation (n = 36). Respondents were allowed to select multiple factors.

A student’s career plans for after graduation provided the strongest prediction for whether a student will desire to stay in the U.S. (Fig. 3). Students who intend to pursue a career outside of academia and research have a 90% probability of pursuing a career path in the U.S. upon graduation. In contrast, students who plan to remain in academic research and believe that they will be treated much better by colleagues back home had an 86% probability of leaving the U.S. upon graduation. For students who wanted to remain in a research-oriented career, who believed that they will not receive overwhelmingly better treatment by colleagues back home, and who did not think that there are better job opportunities in the U.S., their plans to remain or leave the U.S. depended on their perception of the quality of their current U.S. advisor. For those who felt their U.S. advisor is better than one they would have had in their home country, there was an 80% probability that they will leave the U.S. In contrast, students who did not believe they have a better advisor in the U.S. compared to that of their home country had a 75% probability of staying in the U.S. Students who want to remain in research, who do not believe they will receive overwhelmingly better treatment upon returning to their home country, who believe there are better job opportunities in the U.S. and have adjusted well to the educational culture in America had an 83% probability that they will remain in the U.S. And lastly, for students who want to remain in research, who do not believe they will receive overwhelmingly better treatment upon returning to their home country, who believe there are better job opportunities in the U.S., and who have not adjusted well to the U.S. educational culture, their decision to remain in the U.S. depended on their perception of their professional network. Students who believed that their U.S. education offered them a better professional network compared to that of their home country had an 83% probability that they will leave the U.S. upon graduation. In contrast, there was a 100% probability of staying in the U.S. among students who did not believe that their U.S. education offered them access to a better professional network. Seventy-eight percent of students indicated that they hoped to remain in the U.S. upon graduation. Among these, professional factors accounted for three of the top four reasons of why respondents wanted to stay in the U.S. (Fig. 4A). Aside from overall quality of life, fewer than 20% of respondents indicated that social, cultural, and personal factors influenced their decisions to stay. Among students who wish to leave the U.S. after graduation (N = 36), family was the number one reason for wishing to leave and accounted for 72% of all respondents (Fig. 4B).

### Foreign programs to attract expatriates

We identified 18 countries with programs to attract expatriates who have studied and are currently living abroad back to their home countries (Table 1). Most programs are open to scientists and researchers of all nationalities but many programs state within their mission statements that a major objective is to reverse the brain drain of its specific country by bringing back their own citizens from abroad (*e.g*., Argentina’s Raices, Bavaria’s Return to Bavaria, Israel’s Gvahim).

## Discussion

A student’s career path after graduation was the most important factor in determining whether he or she will remain in the U.S. or return to his or her home country. Students who wished to pursue a career in industry or a non-governmental organization (NGO) overwhelmingly decided to remain in the U.S. upon graduation. This suggests that despite recent economic woes, the U.S. is still perceived as an attractive country for scientists and researchers, which was echoed in our interviews. “It is quite likely that I will end up in the U.S. because it is still seen as the center of innovation and the best opportunities will be in the U.S.” stated a Mechanical Engineering graduate student. A graduate student from Chemical Engineering commented that the reason he chose to come to the U.S. for his graduate studies was because he can receive a good education in the U.S. and that “research is more advanced here in the U.S.” Students also indicated that a major difference between the U.S. education system and that of their home countries is the emphasis of critical thinking and openness in classroom discussions in the U.S. A Geography graduate student commented that her “biggest challenge [to living in the U.S.] was to adapt to critical thinking. My background knowledge is very strong because of my education [but critical thinking] is something I had to work on.”

For those who wish to work in industry or a NGO, we found that if given a choice, 90% of would choose to remain in the U.S. after graduation. Past studies have shown that, in fact, the percentage of foreign S&E doctorate recipients staying in the U.S. is approximately 50% [37, 38]. Although this percentage is based on scientists from all career paths, it perhaps provides a rough estimate for how difficult it is for a foreign born scientist to remain in the U.S. after graduation, suggesting that many highly skilled foreign scientists and engineers who wish to remain in the U.S. may be forced to leave. There are many factors that lead to this observation, and, notably, U.S. immigration policy plays a key role.

The predominant method for foreign citizens to remain and work in the U.S. currently is through the H-1B visa program, in which foreign workers are sponsored by U.S. businesses. The H-1B visa has been widely criticized for having high rejection rates, low caps, and contributing to the large exodus of highly skilled immigrant workers leaving the U.S. [5]. For the H-1B fiscal year (FY) 2015 “cap season,” which began on April 1, 2014, a regular cap of 65,000 H-1B visas, and an exemption of 20,000 H-1B visas for individuals who have obtained a U.S. master’s degree or higher, were mandated [39]. For the FY 2014 cap season, the H-1B visas were capped at 65,000 visas total, with no exemptions for higher degree recipients. The FY 2014 cap filled within the first week of the filing period. During this time, the U.S. Citizenship and Immigration Services office received approximately 124,000 H-1B petitions [39].

Students recognize the limitations imposed by these policies. “The H-1 visa makes you get a sponsor for 5 years or so and you are bound to that employer and that is not very attractive. If the U.S. wants to retain talent, people need freedom to pursue what they want to research,” stated an Electrical and Computer Engineering graduate student. A Mechanical Engineering graduate student from a country with poor relations with the U.S. stated the frustration felt by many international students: “The fact that you don’t have a green card at the end of your PhD—it’s a nightmare. For international students, not having a green card, it impacts the job search, everything. The U.S. is welcoming to graduate students to come and study but there doesn’t seem to be a plan for after students graduate. Students settle for jobs that are below them because they work for companies that will provide them with a green card.”

Studies have shown that foreign scientists and entrepreneurs play an important role in the U.S. economy because they not only help create new businesses and jobs, but are also a key source of American innovation: foreign-born scientists and engineers contribute to more than half of the international patents filed by U.S. based multinational corporations (for a review, see [5]). Our study suggests that changes in the current U.S. immigration policy regarding PhD graduates in STEM fields are needed if the U.S. wants to retain the talent that it has helped create. American policymakers are aware of the importance in retaining foreign scientists who have been trained in the U.S. Most recently, U.S. lawmakers proposed legislation known as the ‘Stopping Trained in America PhDs from Leaving the Economy Act of 2011’ (*i.e*., STAPLE Act) to exempt PhD STEM degree holders who graduated from a U.S. institution of higher education from the numerical limitations of the H-1B visa and to be admitted for permanent residence (*i.e*., green card) went before Congress in January 2011 [40]. The act did not pass the 112^th^ Congress (2011–2012) and was reintroduced on March 2013 to the 113^th^ Congress (2013–2014) [41]. The bill, if passed, would exempt foreign-born individuals who have a U.S. STEM PhD diploma from the H-1B numerical limitations. The potential economic impact that foreign-born, U.S. trained scientists can have on a country has been recognized by many source countries (Table 1). These countries have created incentive programs in the hopes of luring highly skilled students who have been educated abroad back to their home countries. The effectiveness of these programs, however, is unclear.

Past studies focused on how single push-pull factors could influence a person’s decision to stay or leave the U.S., but did not examine how these factors interacted with one another [22]. Our results suggest that for students who are interested in remaining in academia or working for a governmental agency after graduation, the interaction of the factors that influenced their decisions to stay or depart the U.S. were much more complex. We found that a person’s decision to stay or leave the U.S. upon graduation was dependent on the interaction of professional (*i.e*., job opportunities, quality of advisor/mentorship, and quality of professional network), personal (*i.e*., perception of treatment by colleagues in their home country), and social/cultural factors (*i.e*., adjustment to American educational culture). Despite having a small sample size, our study provides a first look at how these factors interact and the implications behind these interactions.

Our results indicate that for those who do not wish to enter industry, perception of how they will be treated by colleagues in their home country is a major factor in determining whether they desire to stay or leave the U.S. upon graduation. Institutional prestige has often been cited as a potential influential factor in driving academic and scientific mobility [42, 43] but few studies have closely examined the roles of personal prestige and preferential treatments as motivating factors of mobility [44, 45]. Our study suggests that as countries develop policies to attract highly skilled researchers, a sense of heightened personal prestige and preferential treatments offered by these policies may play a larger role in driving scientific mobility than in previous decades. Past research has found that individuals who return to China after building a career in the U.S. are offered considerable tax and housing incentives and startup or research funding, which can kickstart their career [46, 47]. Nonetheless, some individuals have expressed concern that they are resented by colleagues who never left China and who were not afforded the same opportunities [46, 47]. It is not clear how aware of such factors students are upon graduating, but potential personal prestige and resentment appear to weigh on them.

For those who did not believe they will be treated significantly better if they returned to their home country, and also believed that the U.S. will not offer better job opportunities, we found, seemingly paradoxically, that those students who believed they had received better mentorship or advising in the U.S. than they would have received in their home country were more likely to leave the U.S., while those who received worse mentorship or advising were more likely to stay. We hypothesize this reflects the greater confidence possessed by students with top U.S. advisors, making them less hesitant about leaving their current networks to pursue independent careers. During our interviews, those respondents who appeared more confident and assertive cited excellent mentorship as a strength of the U.S. academic system. On the other hand, we theorize that students who did not receive effective mentorship from their advisors would be less willing to leave their current professional networks to return to their home country, where professional success is often less certain, inadequate funding levels, and institutional support often lacking.

For students who felt that they have not adjusted well to American educational culture, the quality of their professional networks determined whether they will remain or depart the U.S. upon graduation. Students who felt that they have not adjusted well to American educational culture but have developed an excellent professional network in the U.S. are more likely to leave the U.S. than students who also have not adjusted well to American educational culture but who felt they were unable to develop a solid U.S.-based professional network. We consider this to be the case because students with strong U.S. professional ties are often able to leverage their connections once they return home, while students lacking such ties are more hesitant to leave, preferring to remain—even if adjustment to the U.S. has been difficult—until they feel more fully established. Such students might seek to continue to stay in the U.S., where they can further develop the professional networks that will be important for their careers once they return to their home country.

We realize that there may be some limitations to our study because of its small sample size but we believe our results are generalizable to the larger international student population in the U.S. because of how closely the demographic distribution of our survey respondents matches to that of the national distribution of international students studying in the U.S. Differences among students of different nationalities were not detectable because of the small sample size and future studies should focus on elucidating whether students from different nationalities are influenced by different factors to pursue their studies in the U.S. and in their decisions to stay or return to their home countries.

## Conclusion

Overall, our survey and interviews suggest that the American university system is still viewed as a world-class destination for international students to train and gain experience in graduate level science and engineering. As a result, the American university system continues to attract some of the world’s top technical talent, thus remaining a beneficiary of the high level of skills and unique perspective offered by the world’s top international students. While our study was performed at a single institution, we received a clear impression that the international graduate student population is composed of a highly motivated and talented group of individuals that are adding substantial value to the university environment, both through providing the local academic community with valuable direct connections to international professional networks as well as bringing different viewpoints to bear on complex problems.

We conclude that a major reason the U.S. academic system remains at the forefront of the world’s scientific communities is because the U.S. system remains so inclusive to the diverse, talented international students who are seeking to pursue educational opportunities outside of their home countries. The United States was home to 28% of all globally mobile students in 2001 and 19% of all globally mobile students in 2012 [48]. The decline in percent share of globally mobile students coming to the U.S. is likely due to multitude of reasons not limited to increased effort put into recruiting foreign students by key competitive nations, immigration-friendly visa policies by other countries, and hesitancy of applying to U.S. institutions due to changing governmental regulations [49, 50]. Despite the decrease in the global share of international students, the U.S. remains the number one destination for students [48]. We therefore find it important to conclude by noting that our interviews brought up two recurring themes that may have direct relevance to the continued excellence of the U.S. university system:

Many students expressed concern about ways in which the complex nature of America’s immigration policies hinders their ability to succeed. In particular, uncertainties about obtaining green cards following graduation were listed as a deterrent for choosing to study in the U.S. and attempting to stay following graduation.

Many students also noted that the U.S. is no longer an automatic choice for obtaining the best PhD education in science and engineering. In particular, Europe was listed as becoming increasingly competitive choice for many students and their undergraduate colleagues. One cause of this is the EU’s relaxed immigration policies, under which students from EU Member States have the opportunity to study at institutions in other EU countries. With cost and proximity so important to students from Asia, why go all the way the U.S.?

Both of these themes show that policy makers can no longer safely assume that the U.S. university system will attract the world’s top talent simply by the virtue of being the world’s most highly desired academic destination. If the U.S. wishes to continue to both attract and keep the world’s best young scientific minds, policy makers must make changes to the current immigration policies regarding advanced degree STEM holders. Universities in other countries are seen as increasing in scientific competitiveness, and as a result the U.S. may lose out to other regions in attracting scientists in the global talent pool. This, in turn, could compromise America’s leading position in research and innovation.

While the U.S. clearly pays a price when the best post-graduates repatriate to their home countries, the loss of talent is not necessarily total. Students who return home often become part of a global innovation network, continuing to work with their colleagues in the U.S. (and elsewhere), encouraging their own students to attend school in the U.S., and contributing to global innovation in which the U.S. plays the major role and reaps many benefits. As Luo and Wang (2002) demonstrate, the migration of talent can create networks of expatriates and returnees who work together to conduct research or build businesses in both countries.

Today, with the rise of China, India, and other emerging economies, there is growing concern in policy circles that the U.S. may be losing its competitive edge [51–57]. Our research strongly suggests that the U.S. is losing out in terms of retaining talented foreign students, in large part because U.S. immigration policies make it difficult for the best and the brightest to remain after graduating, even though the large majority would prefer to do so. While some returnees may retain their ties with former U.S. professors and colleagues, it is a matter of debate whether this offsets the direct loss of talent through repatriation. We argue that by reworking immigration policies and thereby making the environment more appealing for the most talented international students to stay for the early portion of their careers, the U.S. would benefit greatly.

## Supporting Information

S1 Summary Total number of survey respondents and percent of total response by country of origin. Countries are listed in order by descending total number of respondents.
(DOCX)

S1 Survey Full survey administered to University of California, Santa Barbara international STEM graduate students during May 2–24, 2013. (DOC)
