# Supplementary material for: Will They Stay or Will They Go? International Graduate Students and Their Decisions to Stay or Leave the U.S. upon Graduation
Source: PLoS One. 2015 Mar 11;10(3):e0118183. doi: 10.1371/journal.pone.0118183 (PMC4356591; doi:10.1371/journal.pone.0118183)
Supplement: S1 Summary — Countries are listed in order by descending total number of respondents. (DOCX) [file pone.0118183.s001.docx]

| **Country of origin** | **Number of respondents** | **Percent of total response (%)** |
| --- | --- | --- |
| China | 47 | 28.3 |
| India | 40 | 24.1 |
| Taiwan | 12 | 7.2 |
| South Korea | 8 | 4.8 |
| Iran | 7 | 4.2 |
| Turkey | 7 | 4.2 |
| Canada | 4 | 2.4 |
| Germany | 4 | 2.4 |
| Italy | 4 | 2.4 |
| Vietnam | 4 | 2.4 |
| Brazil | 3 | 1.8 |
| Thailand | 3 | 1.8 |
| Chile | 2 | 1.2 |
| Mexico | 2 | 1.2 |
| United Kingdom | 2 | 1.2 |
| Belgium | 1 | 0.6 |
| Cyprus | 1 | 0.6 |
| Denmark | 1 | 0.6 |
| France | 1 | 0.6 |
| Greece | 1 | 0.6 |
| Hong Kong | 1 | 0.6 |
| Kenya | 1 | 0.6 |
| Malaysia | 1 | 0.6 |
| Moldova | 1 | 0.6 |
| New Zealand | 1 | 0.6 |
| Norway | 1 | 0.6 |
| Peru | 1 | 0.6 |
| Romania | 1 | 0.6 |
| Russia | 1 | 0.6 |
| Saudi Arabia | 1 | 0.6 |
| Sri Lanka | 1 | 0.6 |
| Ukraine | 1 | 0.6 |
| **Total** | **166** | **100** |
