# Supplementary material for: Will They Stay or Will They Go? International Graduate Students and Their Decisions to Stay or Leave the U.S. upon Graduation
Source: PLoS One. 2015 Mar 11;10(3):e0118183. doi: 10.1371/journal.pone.0118183 (PMC4356591; doi:10.1371/journal.pone.0118183)
Supplement: S1 Survey — (DOC) [file pone.0118183.s002.doc]

**International Students in Science Survey**

***Use the dropdown menu above if you would like to take this survey in Chinese.***

You are being asked to participate in a short survey for a research study about your decision to live and study in the USA. We will also ask you about your future career aspirations. Reports from this survey will be shared in academic publications and conferences.

We are conducting a study of international graduate students in the physical and life sciences to determine their reasons for choosing study in the United States, their educational background, and their future plans. This is part of a larger set of research that compares the national innovation capacity of the United States with its peers in Asia, Latin America, and Europe. The United States is a primary destination for some of the world's brightest scientific minds. Once

complete, the findings will help inform a nationwide study asking similar questions.

While investigators will have access to individual level data, reports will be shared only in the aggregate, making any identification unlikely. There are no other foreseeable risks. Your participation is entirely voluntary. The survey will last approximately 10 minutes. The survey includes questions about family income, geographical origin, test scores and your career aspirations.

Your response is confidential, and will not be associated in any way with your identity. Summary of results may be presented to a wider public in the form of future presentations and publications, however data will be reported only in the aggregate. Your contact information will be used for raffle prize assignment, and will not be associated with your response.

If you complete this survey you will be entered in a raffle to win one of two $25 UCSB Bookstore Gift Certificates, one of two $10 UCSB Bookstore Gift Certificates, one of two $10 Jamba Juice gift cards, four of sixteen gift certificates to Zodo's Bowling, one Santa Barbara Axxess book, or one gift bag of Trader Joe's goodies. We estimate your odds to be 1 in 50. You are eligible even if you skip questions or withdraw from the study.

There are no right or wrong answers. You are free to skip any question you do not wish to answer, and you may terminate the survey and your participation in the study at any time. You may refuse to participate and still receive any benefits you would receive if you were not in the study. You may change your mind about being in the study and quit after the study has started.

If you have any questions about this research project or if you think you may have been injured as a result of your

participation, please contact: [removed author information]

If you have any questions regarding your rights and participation as a research subject, please contact the Human Subjects Committee at (805) 893-3807 or hsc@research.ucsb.edu. Or write to the University of California, Human Subjects Committee, Office of Research, Santa Barbara, CA 93106-2050

There are 51 questions in this survey.

**1. Did you study in the US for any part of your undergraduate**

**degree?**

Please choose **only one** of the following:

· Yes

· No

**2. How long did you study at an American university during**

**your undergraduate career?**

Please choose **only one** of the following:

· Less than 1 year

· 1 year

· 1-2 years

· 3+ years

**3. Did you receive your undergraduate degree in the U.S.?**

Please choose **only one** of the following:

· Yes

· No

**4. What university did you complete your undergraduate**

**degree at?**

Please write your answer here:

**5. If you completed any other degrees, what degrees did you**

**complete and at which universities did you do so?**

Please write your answer here:

**6. Did you take the SAT?**

Please choose **only one** of the following:

· Yes

· No

**7. What did you score in the following subsections? (If you**

**do not remember, please enter 0)**

Please write your answer(s) here:

· Math:

· Verbal:

**[7B]**

· Writing:

**8. What did you score on the Graduate Record Examination**

**(GRE)? (If you do not remember, please enter 0)**

Please write your answer(s) here:

· Math:

· Verbal:

**[8B]**

Each answer must be between 0 and 6

Please write your answer here:

· Writing:

**2**

**9. How did you apply to the graduate program at UCSB?**

Please choose **only one** of the following:

· Applied through an agency

· Applied through a governmental program in your home country

· Self-application

· Transferred to UCSB from another graduate program in the U.S.

· Other (please specify):

**10. What factors influenced your decision to do your**

**graduate studies in the US? (Please check all that apply)**

Please choose **all** that apply:

· Higher quality of education

· Lower cost

· Opportunity to work with specific faculty

· Future career opportunities

· Wanted to live in the United States

· Proximity to friends / family

· Wanted to experience living abroad

· Other (please specify): :

**11. What are your plans after graduation?**

Please choose **only one** of the following:

· Continue conducting research and/or teach through a post-doc or other academic position

· Work for a government

· Work for non-governmental organization

· Seek employment with a company

· Start a company

· Other (please specify):

**12. You told us in the previous question that you plan to**

**conduct research after graduation. In which country do you**

**plan to do so?**

Please choose **only one** of the following:

· Afghanistan

· Albania

· Algeria

· Andorra

· Angola

· Anguilla

· Antigua

· Argentina

· Armenia

· Aruba

· Australia

· Austria

· Azerbaijan

· Bahamas

· Bahrain

· Bangladesh

· Barbados

· Belarus

· Belgium

· Belize

· Benin

· Bermuda

· Bhutan

· Bolivia

· Bosnia & Herzegovina

· Botswana

· Brazil

· British Virgin Islands

· Brunei

· Bulgaria

· Burkina Faso

· Burundi

· Cambodia

· Cameroon

· Canada

· Cape Verde

· Cayman Islands

· Central African Republic

· Chad

· Chile

· China

· Colombia

· Comoros

· Congo

· Cook Islands

· Costa Rica

· Côte d’Ivoire

· Croatia

· Cuba

· Cyprus

· Czech Republic

· Denmark

· Djibouti

· Dominica

· Dominican Republic

· Ecuador

· Egypt

· El Salvador

· Equatorial Guinea

· Eritrea

· Estonia

· Ethiopia

· Falkland Islands

· Fed States of Micronesia

· Fiji

· Finland

· France

· French Guiana

· French Polynesia

· Gabon

· Gambia

· Georgia

· Germany

· Ghana

· Gibraltar

· Greece

· Grenada

· Guadeloupe

· Guatemala

· Guinea

· Guinea-Bissau

· Guyana

· Haiti

· Honduras

· Hong Kong

· Hungary

· Iceland

· India

· Indonesia

· Iran

· Iraq

· Ireland

· Israel

· Italy

· Jamaica

· Japan

· Jordan

· Kazakhstan

· Kenya

· Kiribati

· Korea Dem People’s Rep

· Kuwait

· Kyrgyzstan

· Laos

· Latvia

· Lebanon

· Leeward Islands

· Lesotho

· Liberia

· Libya

· Liechtenstein

· Lithuania

· Luxembourg

· Macao

· Macedonia

· Madagascar

· Malawi

· Malaysia

· Maldives

· Mali

· Malta

· Marshall Islands

· Martinique

· Mauritania

· Mauritius

· Mexico

· Moldova

· Monaco

· Mongolia

· Montserrat

· Morocco

· Mozambique

· Myanmar

· Namibia

· Nauru

· Nepal

· Netherlands

· Netherlands Antilles

· New Caledonia

· New Zealand

· Nicaragua

· Niger

· Nigeria

· Niue

· Norway

· Oman

· Pakistan

· Palau

· Palestinian Authority

· Panama

· Papua New Guinea

· Paraguay

· Peru

· Philippines

· Poland

· Portugal

· Qatar

· Republic of Korea

· Reunion

· Romania

· Russia

· Rwanda

· San Marino

· São Tomé & Príncipe

· Saudi Arabia

· Senegal

· Seychelles

· Sierra Leone

· Singapore

· Slovakia

· Slovenia

· Solomon Islands

· Somalia

· South Africa

· South America

· Spain

· Sri Lanka

· St Kitts-Nevis

· St Lucia

· St Vincent

· Sudan

· Suriname

· Swaziland

· Sweden

· Switzerland

· Syria

· Taiwan

· Tajikistan

· Tanzania

· Thailand

· Togo

· Tonga

· Trinidad & Tobago

· Tunisia

· Turkey

· Turkmenistan

· Turks & Caicos Islands

· Tuvalu

· Uganda

· Ukraine

· United Arab Emirates

· United Kingdom

· United States

· Uruguay

· USSR Former

· Uzbekistan

· Vanuatu

· Vatican City

· Venezuela

· Vietnam

· Western Sahara

· Western Samoa

· Windward Islands

· Yemen

· Yugoslavia Former

· Zaire/Congo

· Zambia

· Zimbabwe

**13. Do you hope to remain in the U.S. after graduation?**

Please choose **only one** of the following:

· Yes

· No

**14A. Why do you want to stay in the US? Select all that**

**apply:**

**Only answer this question if the following conditions are met:**

° ((**13.NAOK** == "1"))

Please choose **all** that apply:

· Job opportunities for myself

· Opportunities for family members

· Salary

· Overall quality of life

· Geographic location

· Family

· Friends

· Professional network

· Cultural reasons

· Social reasons

· Other (please specify): :

**15A. How long do you plan on staying in the US?**

Please choose **only one** of the following:

· 0-2 years

· 3-4 years

· 4-6 years

· 6-8 years

· 8-10 years

· >10 years

· Don’t know/Not sure

**14B. Why do you wish to leave the US? Select all that apply:**

Please choose **all** that apply:

· Job opportunities for myself

· Opportunities for family members

· Salary

· Overall quality of life

· Geographic location

· Family

· Friends

· Professional network

· Cultural reasons

· Social reasons

· Other (please specify): :

**15B. Do you know which country you plan to go after**

**graduation?**

Please choose **only one** of the following:

· Yes

· No/Not sure

**16. Please select which country you plan to go after**

**graduation:**

Please choose **only one** of the following:

· Afghanistan

· Albania

· Algeria

· Andorra

· Angola

· Anguilla

· Antigua

· Argentina

· Armenia

· Aruba

· Australia

· Austria

· Azerbaijan

· Bahamas

· Bahrain

· Bangladesh

· Barbados

· Belarus

· Belgium

· Belize

· Benin

· Bermuda

· Bhutan

· Bolivia

· Bosnia & Herzegovina

· Botswana

· Brazil

· British Virgin Islands

· Brunei

· Bulgaria

· Burkina Faso

· Burundi

· Cambodia

· Cameroon

· Canada

· Cape Verde

· Cayman Islands

· Central African Republic

· Chad

· Chile

· China

· Colombia

· Comoros

· Congo

· Cook Islands

· Costa Rica

· Côte d’Ivoire

· Croatia

· Cuba

· Cyprus

· Czech Republic

· Denmark

· Djibouti

· Dominica

· Dominican Republic

· Ecuador

· Egypt

· El Salvador

· Equatorial Guinea

· Eritrea

· Estonia

· Ethiopia

· Falkland Islands

· Fed States of Micronesia

· Fiji

· Finland

· France

· French Guiana

· French Polynesia

· Gabon

· Gambia

· Georgia

· Germany

· Ghana

· Gibraltar

· Greece

· Grenada

· Guadeloupe

· Guatemala

· Guinea

· Guinea-Bissau

· Guyana

· Haiti

· Honduras

· Hong Kong

· Hungary

· Iceland

· India

· Indonesia

· Iran

· Iraq

· Ireland

· Israel

· Italy

· Jamaica

· Japan

· Jordan

· Kazakhstan

· Kenya

· Kiribati

· Korea Dem People’s Rep

· Kuwait

· Kyrgyzstan

· Laos

· Latvia

· Lebanon

· Leeward Islands

· Lesotho

· Liberia

· Libya

· Liechtenstein

· Lithuania

· Luxembourg

· Macao

· Macedonia

· Madagascar

· Malawi

· Malaysia

· Maldives

· Mali

· Malta

· Marshall Islands

· Martinique

· Mauritania

· Mauritius

· Mexico

· Moldova

· Monaco

· Mongolia

· Montserrat

· Morocco

· Mozambique

· Myanmar

· Namibia

· Nauru

· Nepal

· Netherlands

· Netherlands Antilles

· New Caledonia

· New Zealand

· Nicaragua

· Niger

· Nigeria

· Niue

· Norway

· Oman

· Pakistan

· Palau

· Palestinian Authority

· Panama

· Papua New Guinea

· Paraguay

· Peru

· Philippines

· Poland

· Portugal

· Qatar

· Republic of Korea

· Reunion

· Romania

· Russia

· Rwanda

· San Marino

· São Tomé & Príncipe

· Saudi Arabia

· Senegal

· Seychelles

· Sierra Leone

· Singapore

· Slovakia

· Slovenia

· Solomon Islands

· Somalia

· South Africa

· South America

· Spain

· Sri Lanka

· St Kitts-Nevis

· St Lucia

· St Vincent

· Sudan

· Suriname

· Swaziland

· Sweden

· Switzerland

· Syria

· Taiwan

· Tajikistan

· Tanzania

· Thailand

· Togo

· Tonga

· Trinidad & Tobago

· Tunisia

· Turkey

· Turkmenistan

· Turks & Caicos Islands

· Tuvalu

· Uganda

· Ukraine

· United Arab Emirates

· United Kingdom

· Uruguay

· USSR Former

· Uzbekistan

· Vanuatu

· Vatican City

· Venezuela

· Vietnam

· Western Sahara

· Western Samoa

· Windward Islands

· Yemen

· Yugoslavia Former

· Zaire/Congo

· Zambia

· Zimbabwe

**3**

**17. Will your US education give you any advantages in your**

**career?**

Please choose the appropriate response for each item:

Strong

disadvantage

(1) (2) (3) (4)

Strong

advantage

(5)

**18. In comparison with your home country, what**

**advantages, if any, do you feel a US education provides?**

**Select all that apply.**

Please choose **all** that apply:

· Better education/knowledge of your field

· Better advisors/mentorship

· Better professional network

· Better job opportunity

· None

· Other (please specify): :

**19. Are you aware of any programs, incentives, or**

**opportunities that are provided by your home country that**

**are intended to encourage you to return after you get your**

**degree?**

Please choose **only one** of the following:

· Yes

· No

**20. What kinds of programs, incentives, or opportunities are**

**you familiar with? Please briefly list:**

Please write your answer here:

**21. Please list any programs you have considered.**

Please write your answer here:

**22. With regard to your academic experience, how would you**

**rate each of the following in comparison with your home**

**country:**

Please choose the appropriate response for each item:

Very

much

worse

(1) (2) (3) (4)

Very

much

better

(5) N/A

Open classroom

discussions

Professors’ teaching

styles

Subject teaching matter

Access to books,

magazines, journals and

databases

Freedom to openly debate

established theories

Freedom to pursue new,

self-proposed research

directions

Collaboration with other

grad students in your lab

**4**

**23. How do you feel you are treated by your colleagues and**

**professors in the US?**

Please choose the appropriate response for each item:

Treated much

worse

(1) (2) (3) (4)

Treated much

better

(5)

**24. How do you feel you would be treated by your colleagues**

**and professors in your home country if you returned?**

Please choose the appropriate response for each item:

Treated much

worse

(1) (2) (3) (4)

Treated much

better

(5)

**25. How successfully do you feel you have adjusted to**

**American educational culture?**

Please choose the appropriate response for each item:

Adjusted

poorly

(1) (2) (3) (4)

Adjusted

completely

(5)

**26. Please select any challenges you may have encountered**

**while adjusting (select all that apply):**

Please choose **all** that apply:

· Cultural challenges

· Social challenges

· Academic challenges

· Racial challenges

· Financial challenges

· Other (please specify): :

**Demographics**

**27. What is your age?**

Each answer must be between 18 and 99

Please write your answer here:

**28. What is your gender?**

Please choose **only one** of the following:

· Female

· Male

**29. What degree are you currently pursuing at UCSB?**

Please choose **only one** of the following:

· Master’s level

· PhD level

**30. Have you advanced to candidacy?**

Please choose **only one** of the following:

· Yes

· No

**31. What year are you in your graduate studies?**

Please choose **only one** of the following:

· 1st year

· 2nd year

· 3rd year

· 4th year

· 5th year

· 6th year

· > 6 years

**32. What field is your father employed in?**

Please choose **only one** of the following:

· Business

· Research

· Management

· Other white collar

· Agriculture

· Education

· Other blue collar

· N/A

**33. What is the highest level of education that your father**

**received?**

Please choose **only one** of the following:

· Up to high school

· High school graduate

· Some college

· Bachelor’s degree

· Professional degree (e.g., MA, JD)

· PhD

· N/A

**34. What field is your mother employed in?**

Please choose **only one** of the following:

· Business

· Research

· Management

· Other white collar

· Agriculture

· Education

· Other blue collar

· N/A

**35. What is the highest level of education that your mother**

**received?**

Please choose **only one** of the following:

· Up to high school

· High school graduate

· Some college

· Bachelor’s degree

· Professional degree (e.g., MA, JD)

· PhD

· N/A

**36. What was your age when you first entered the United**

**States for education?**

Each answer must be between 1 and 99

Please write your answer here:

·

**Chinese Student Questions**

**37. Did you take the Chinese College Entrance Exam**

**(Gaokao)?**

Please choose **only one** of the following:

· Yes

· No

**38. What year did you take the Gaokao?**

Each answer must be between 1900 and 2013

Please write your answer here:

·

**39. What was your TOTAL score? (If you do not remember, please**

**enter 0)**

Each answer must be between 0 and 750

Please write your answer here:

· Score:

**40. What did you score in each of the following**

**subsections? (If you do not remember, please enter 0)**

Please write your answer(s) here:

· Chinese:

· Mathematics:

· English:

**41. Did you take the TOEFL?**

Please choose **only one** of the following:

· Yes

· No

**42. What were your scores in the following sections? (If you do**

**not remember, please enter 0)**

Please write your answer(s) here:

· Reading:

· Listening:

· Speaking:

· Writing:

**Follow Up**

**We would welcome the opportunity to have a brief follow-up**

**interview. Please let us know if you are willing to do so.**

Please choose **only one** of the following:

· Yes

· No

**Contact Form**

**[Name] ***

Please write your answer(s) here:

· First name:

· Last name:

**[Phone] ***

Please check the format of your answer.

Please write your answer here:

**50 [Email] ***

Please check the format of your answer.

Please write your answer here:

**[Contact]**

**Preferred mode of contact: ***

Please choose **only one** of the following:

· Phone

· Email
